# Crystal structure of RahU, an aegerolysin protein from the human pathogen *Pseudomonas aeruginosa*, and its interaction with membrane ceramide phosphorylethanolamine

**DOI:** 10.1038/s41598-021-85956-2

**Published:** 2021-03-22

**Authors:** Eva Kočar, Tea Lenarčič, Vesna Hodnik, Anastasija Panevska, Yunjie Huang, Gregor Bajc, Rok Kostanjšek, Anjaparavanda P. Naren, Peter Maček, Gregor Anderluh, Kristina Sepčić, Marjetka Podobnik, Matej Butala

**Affiliations:** 1 Department of Biology, Biotechnical Faculty, University of Ljubljana, 1000 Ljubljana, Slovenia; 2 Department of Molecular Biology and Nanobiotechnology, National Institute of Chemistry, 1000 Ljubljana, Slovenia; 3 Department of Paediatrics, Division of Pulmonary Medicine, Cincinnati Children’s Hospital Medical Center, Cincinnati, OH 45229 USA; 4 Present Address: Centre for Functional Genomics and Bio-Chips, Institute of Biochemistry and Molecular Genetics, Faculty of Medicine, University of Ljubljana, 1000 Ljubljana, Slovenia

**Keywords:** Microbiology, Structural biology, Lipids, Membrane lipids, Sphingolipids, Biochemistry, Proteins, Membrane proteins, Structural biology, X-ray crystallography

## Abstract

Aegerolysins are proteins produced by bacteria, fungi, plants and protozoa. The most studied fungal aegerolysins share a common property of interacting with membranes enriched with cholesterol in combination with either sphingomyelin or ceramide phosphorylethanolamine (CPE), major sphingolipids in the cell membranes of vertebrates and invertebrates, respectively. However, genome analyses show a particularly high frequency of aegerolysin genes in bacteria, including the pathogenic genera *Pseudomonas* and *Vibrio*; these are human pathogens of high clinical relevance and can thrive in a variety of other species. The knowledge on bacterial aegerolysin-lipid interactions is scarce. We show that *Pseudomonas aeruginosa* aegerolysin RahU interacts with CPE, but not with sphingomyelin-enriched artificial membranes, and that RahU interacts with the insect cell line producing CPE. We report crystal structures of RahU alone and in complex with tris(hydroxymethyl)aminomethane (Tris), which, like the phosphorylethanolamine head group of CPE, contains a primary amine. The RahU structures reveal that the two loops proximal to the amino terminus form a cavity that accommodates Tris, and that the flexibility of these two loops is important for this interaction. We show that Tris interferes with CPE-enriched membranes for binding to RahU, implying on the importance of the ligand cavity between the loops and its proximity in RahU membrane interaction. We further support this by studying the interaction of single amino acid substitution mutants of RahU with the CPE-enriched membranes. Our results thus represent a starting point for a better understanding of the role of *P. aeruginosa* RahU, and possibly other bacterial aegerolysins, in bacterial interactions with other organisms.

## Introduction

Members of the aegerolysin protein family are acidic proteins of 13–20 kDa, which are mainly found in bacteria and fungi. The biological role of these proteins is largely unknown^1^. It has been shown that some of the aegerolysins act as a necessary component of binary pore-forming toxins. The structures of these aegerolysins from the bacteria *Alcaligenes faecalis*^2^ and *Bacillus thuringiensis*^3^, and from the fungus *Pleurotus ostreatus*^4,5^ reveal a packed β-sandwich fold. These aegerolysins bind to membranes of the susceptible cells, where they form lytic complexes in concert with a larger protein partner co-produced by the same organism^2–5^.

While the aegerolysins from *A. faecalis* and *B. thuringiensis* were reported to be associated with (glyco)protein receptors in the midgut of susceptible insects^2,6,7^, the aegerolysins from fungi reported to date act as membrane lipid-binding proteins. Ostreolysin A (OlyA), OlyA6, pleutrotolysin A (PlyA) and PlyA2 from *Pleurotus* species bind specifically to lipid membranes enriched in cholesterol and either sphingomyelin or ceramide phosphorylethanolamine (CPE)^8–11^. Their affinity for cholesterol/CPE membranes is more than 1000 times stronger than for the cholesterol/sphingomyelin membranes^9,12^. Meanwhile, erylysin A (EryA) from *Pleurotus eryngii* and nigerolysin A2 (NigA2) from *Aspergillus niger* interact with CPE/cholesterol-enriched membranes only^9,10,13^.

As mentioned above, *Pleurotus*-derived aegerolysins form lytic bi-component protein complexes in host membranes. Upon binding to CPE- or sphingomyelin/cholesterol-enriched membranes, aegerolysins recruit their 59-kDa protein partners bearing a membrane-attack complex/perforin (MACPF) domain to these membranes, oligomerizing into ring-shaped 13-meric structures. Following that, the MACPF protein partner undergoes large conformational changes and thereby forms a functional pore through the membrane^5,11^.

While sphingomyelin is the major sphingolipid in mammalian cells, its analogue CPE is the principal representative of the sphingosine-based lipids in invertebrates, especially insects^14^. In accordance with these results, recent reports demonstrate that fungal aegerolysins binds insect cells^9,10,13,15^, and permeabilize them in combination with the MACPF protein partner^10^. With this, *Pleurotus*-derived aegerolysins exert insecticidal effects as effective lipid-targeting biopesticides. Aegerolysin orthologues from the bacteria *Clostridium bifermentans* subsp. *malaysia*, *A. faecalis* and *B. thuringiensis* were also described as entomopathogenic and function as binary cytotoxins, or may even be composed of four different subunits^2,16,17^.

An opportunistic human pathogen *P. aeruginosa* is prevalent in patients suffering from cystic fibrosis or acquired immune deficiency syndrome (AIDS)^18^. It is also of importance, however, that *P. aeruginosa* isolates can also infect a variety of evolutionarily different hosts, causing diseases in invertebrates, vertebrates and even plants^19,20^. To succeed in infecting evolutionarily different hosts, *P. aeruginosa* strains have developed an arsenal of virulence factors^21,22^. The expression of these virulence determinants is coordinated by cell density-dependent regulation, for which *P. aeruginosa* employs multiple quorum-sensing systems^23^. We previously showed that when the quorum sensing receptor RhlR forms a complex with its cognate autoinducer *N*-butiryl-homoserine lactone, it triggers the expression of aegerolysin RahU^24^. The *rahU* gene expression is increased about eightfold in the *P. aeruginosa* non-mucoid phenotype isolate, which is associated with the initial phase of lung infection. In comparison, the alginate-overproducing mucoid isolate enables the transition to chronic infection^25^. The biological role of RahU remains puzzling, but it was suggested that it might play a role in host–pathogen interaction^26^. In support of this idea, RahU was shown to be associated with both the inner and outer membrane of the mucoid isolate^25^.

In this work, we show for the first time that the bacterial aegerolysin RahU from *P. aeruginosa* interacts specifically with CPE. This CPE-specific interaction was shown both on the artificial membranes containing physiologically relevant concentrations of CPE, as well as on an insect cell line producing CPE. However, no interaction of RahU could be observed with the mammalian cell membrane. The crystal structures of RahU revealed the conserved aegerolysin fold as well as a ligand binding cavity, harbouring a Tris molecule from the crystallization solution. With further mutational analysis we showed that residues within and surrounding the Tris binding site are important for RahU interaction with CPE containing membranes. Thus, our results provide insights into the molecular discrimination of host cell membranes by RahU.

## Results and discussion

### RahU exhibits binding specificity for CPE in the membranes

Interactions of fungal aegerolysins with artificial and biological membranes have been well studied^1^. Reports reveal that *Pleurotus* aegerolysins OlyA, OlyA6, PlyA2 and EryA as well as the aegerolysin NigA2 from *A. niger*, show a high affinity in the range of 1–10 nM for the membranes consisting of equimolar mixtures of CPE and cholesterol, and OlyA, OlyA6, PlyA and PlyA2 show about 1000 times lower affinity for the equimolar sphingomyelin/cholesterol membrane microdomains^8–11,13,27^. Furthermore, it was shown that OlyA6 and PlyA2 bind to CPE-enriched membranes also in the absence of cholesterol^15^. High-affinity binding to CPE/cholesterol mixtures seems to be a common characteristic of the fungal aegerolysins studied, suggesting their role in the defence against predators^10,13^. On the other hand, data on the binding properties of bacterial aegerolysins are scarce, but they seem to be different than those of fungi. For example, a bacterial orthologue Cry34Ab1 from *B. thuringiensis* interacts only weakly with lipid vesicles containing cholesterol and either CPE, sphingomyelin or 1-palmitoyl-2-oleoyl-*sn*-glycero-3-phosphocholine (POPC)^10^, and insect brush border glycolipids have been reported as its receptors^6,7^. The aegerolysin AfIP-1A from *A. faecalis* was suggested to target the same insect brush border components^2^. The *P. aeruginosa* aegerolysin PA0122 (later renamed as RahU) was reported to interact with oxidized low density lipoproteins and with their major component, lysophosphatidylcholine (lysoPC)^25^. In our previous study, we reproduced the weak interaction between RahU and lysoPC and we also demonstrated a weak interaction of RahU with rhamnolipid biosurfactants^24^, which led us to search further for one or more specific ligands for RahU.

**Figure 1:**
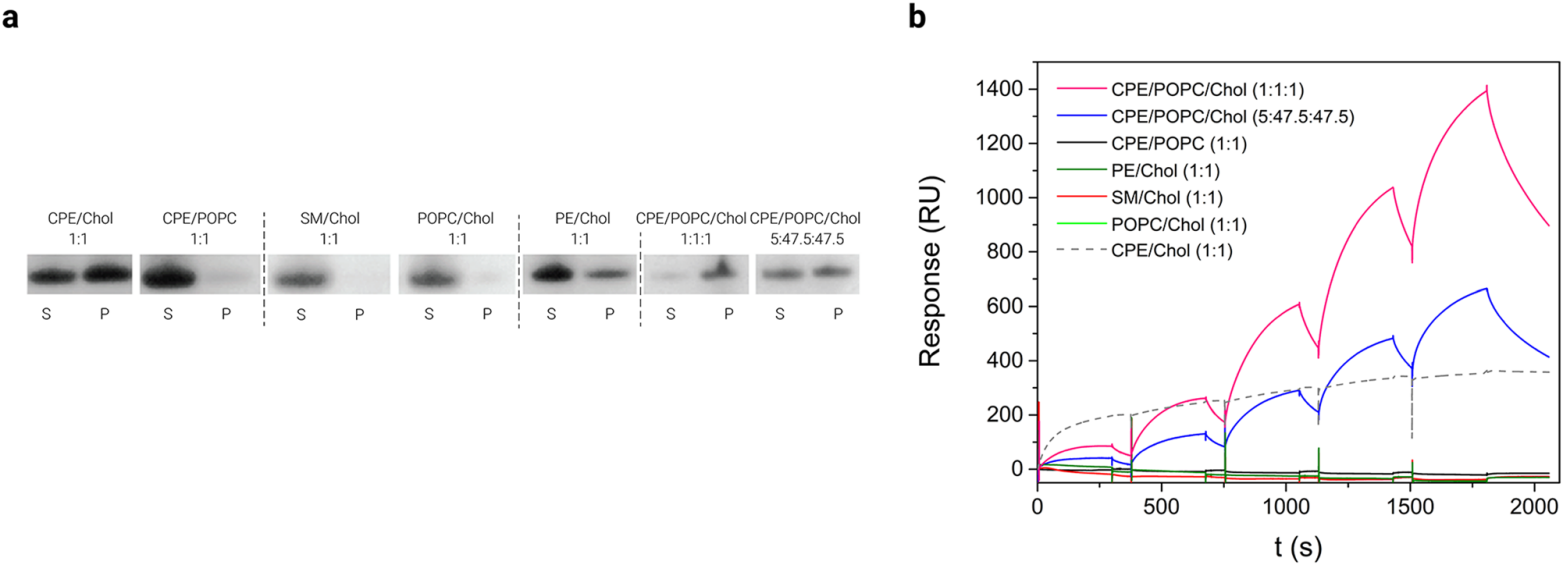
RahU selects for CPE in lipid membranes. (**a**) Binding of RahU protein to multilamellar vesicles with various lipid compositions, as indicated with mole-to-mole ratios. The liposome-RahU co-sedimentation experiments were performed in Hepes buffer pH 7.4. The supernatant (S) and pellet (P) fractions were subjected to SDS-PAGE analysis and stained with Coomassie Blue. The dotted vertical dividing lines indicate the cropped parts of the same gel image. The full-length gels are shown in Fig. S1. (**b**) Surface plasmon resonance analysis of RahU protein in 10 mM Hepes buffer binding to immobilized large unilamellar vesicles, composed of equimolar CPE/POPC, SM/cholesterol, POPC/cholesterol or PE/cholesterol, CPE/cholesterol, CPE/POPC/cholesterol, or 5:47.5:47.5 (mol:mol:mol) CPE/POPC/cholesterol by applying kinetic titration approach via consecutive injections of RahU in 0.5, 1, 2, 3 and 4 μM (from left to right) concentration. Vesicles were immobilized to Biacore L1 chip to 7000–11,000 RU, except for large unilamellar vesicles (LUVs) composed of equimolar CPE/cholesterol, which were immobilized to 1400 RU due to their instability, and set as baseline 0 RU in order to show only the RahU-vesicle interaction (the sensorgram, showing RahU binding to CPE/cholesterol vesicles is shown as a dashed line to indicate that fewer vesicles were immobilized on the chip than for vesicles of other lipid composition). Representative sensorgrams from two independent experiments are shown. *S* supernatant, *P* pellet, *CPE* ceramide phosphorylethanolamine, *Chol* cholesterol, *POPC* 1-palmitoyl-2-oleoyl-sn-glycero-3-phosphocholine, *SM* sphingomyelin, *PE* phosphatidylethanolamine.

We first used a vesicle sedimentation test to study the interaction of RahU isolated either in Hepes or in Tris buffer with multilamellar vesicles (MLVs) of different lipid composition in 10 mM Hepes buffer (pH 7.4) containing 150 mM NaCl (Figs. 1a, S1). We observed RahU in the sediment obtained after centrifugation of the protein mixture with equimolar CPE/cholesterol MLVs, which suggested the RahU association with these lipid membranes. The recovery of RahU in the sediment was even stronger with equimolar CPE/POPC/cholesterol MLVs. We also observed partial co-sedimentation of RahU even with vesicles containing only 5 mol% CPE, which is approximately the molar ratio of CPE detected in membranes of invertebrates^14^. Furthermore, sedimentation experiments suggest that RahU shows even some affinity for the phosphatidylethanolamine (PE)/cholesterol equimolar MLVs, but it did not interact with other vesicles lacking CPE (Figs. 1a, S1). It is noteworthy that PE and CPE contain phosphorylethanolamine as the head group, which is connected with either diacylglycerol or ceramide backbone, respectively. Thus, our results imply that RahU can differentiate between membranes of different lipid composition and that CPE is the preferred target for RahU binding. Since RahU did not interact with CPE/POPC membranes but it did with the CPE/cholesterol ones, these data imply, that co-occurrence of cholesterol enhances RahU binding.

To support the observation that RahU can interact with membranes containing CPE and cholesterol, we performed surface plasmon resonance (SPR) analysis on immobilized on-chip LUVs consisting of equimolar CPE/POPC, SM/cholesterol, POPC/cholesterol, PE/cholesterol, CPE/cholesterol or CPE/POPC/cholesterol, or 5:47.5:47.5 (mol:mol:mol) CPE/POPC/cholesterol (Fig. 1b). It is of note that LUVs of equimolar CPE/cholesterol are unstable, which prevented efficient chip-immobilization of these vesicles. We applied kinetic titration approach by injecting RahU at five concentrations ranging from 0.5 to 4 µM in Hepes buffer successively over the chip-immobilized LUVs^28,29^, without dissociation time between protein injections. The SPR results showed that RahU interacts with CPE-rich membranes (Fig. 1b). The only inconsistency with the data obtained by the co-sedimentation assay was that, when using SPR, we could not detect the interaction of RahU with LUVs of equimolar PE/cholesterol composition (Fig. 1b). Compared to the SPR analysis, we used a sixfold higher concentration of RahU in the co-sedimentation test, and we believe this may be the reason for the different results, i.e. no binding signal in SPR in the case of PE/cholesterol.

### Crystal structures of the *P. aeruginosa* RahU

**Figure 2:**
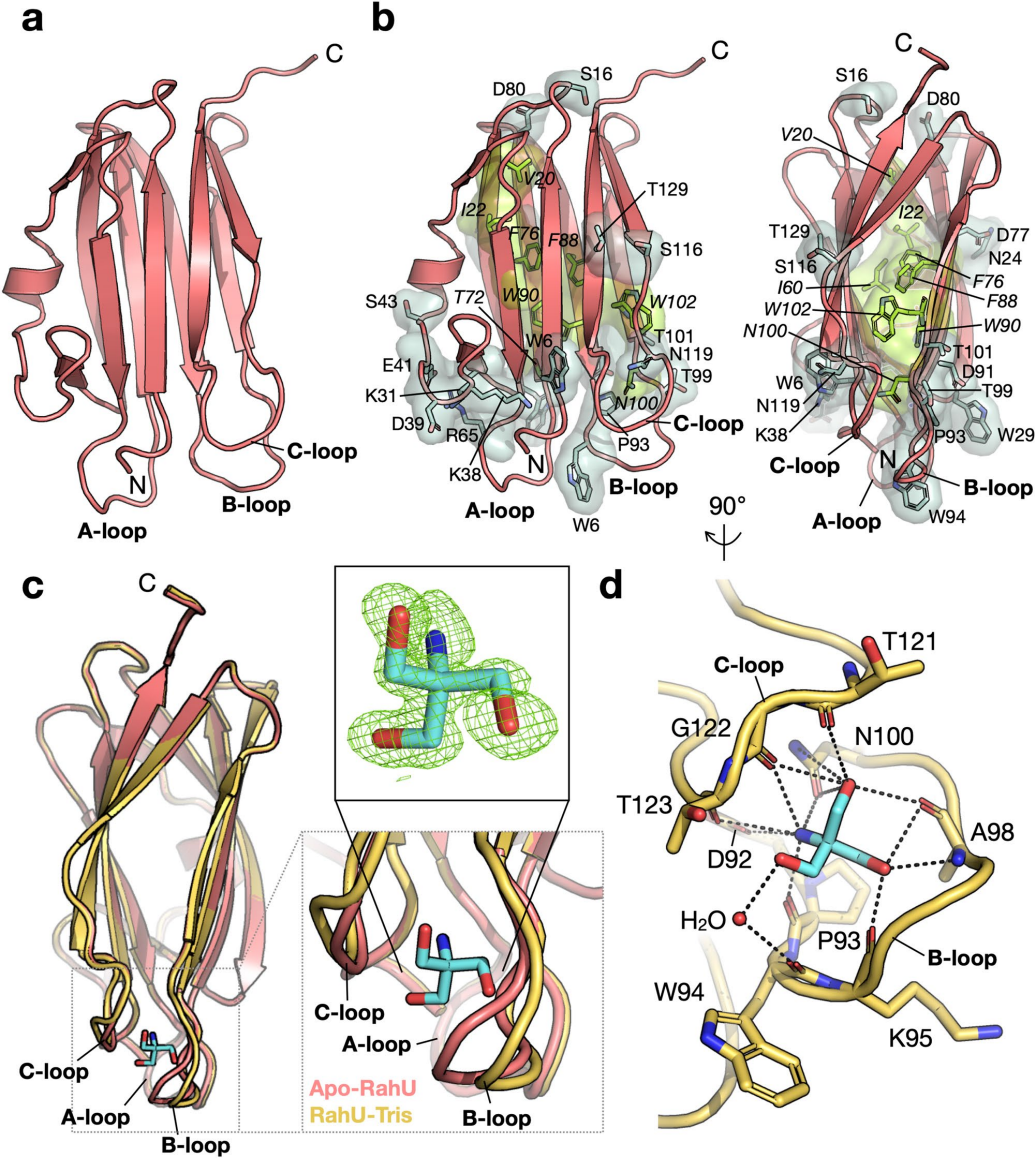
Structural analysis of RahU protein. (**a**) Overall crystal structure of apo-RahU. A-loop, B-loop and C-loop as well as N- and C-termini are indicated. (**b**) Conserved amino acid residues in RahU and fungal aegerolysins OlyA and PlyA are highlighted in sticks. Light green: amino acid residues of the hydrophobic β-sandwich core; light blue: solvent-exposed amino acid residues. Two orientations, 90° relative to each other, are shown. (**c**) Overall crystal structure of RahU (cartoon, yellow) in complex with a Tris molecule (sticks, light blue), compared to apo-RahU (cartoon, red). Insets: Close-up view of conformational differences upon Tris binding, and 3σ contoured Fo-Fc electron density (mesh) before the model of the Tris molecule was added. The model of Tris as positioned in the final refinement cycle is shown in sticks. (**d**) Tris binding-site analysis. Amino acid residues that are involved directly or indirectly via water molecule (shown as a non-bonded sphere) in the Tris binding are highlighted and shown in sticks. Hydrogen bonds with a distance below 3.5 Å between the ligand and amino acid residues are depicted in black dashed lines.

In order to better characterise the RahU protein and its interaction with the host specific lipids, we performed structural studies. Recombinant RahU protein was expressed using *E. coli* expression system, purified and subjected to crystallization trials. It is of note that RahU was purified in 20 mM Tris buffer and formed crystals in 0.2 M di-Sodium tartrate, 20% (w/v) PEG 3350. The crystal structure of the apo-RahU was determined at 1.27 Å resolution (Figs. 2a, S2a and Table S1). The structure shows that RahU is a single domain protein with a distinct β-sandwich architecture that is typical for bacterial and fungal aegerolysins^2–5^ (Fig. S3a). According to Dali superposition based on Cα atoms^30^, RahU fold superimposes better with fungal aegerolysins OlyA from *P. ostreatus*^4^ and PlyA^5^, with root-mean-squared deviations (RMSD) of 1.6 Å and 1.5 Å, respectively, than with bacterial aegerolysins Cry34Ab1 from *B. thuringiensis*^3^ or AfIP-1A from *A. faecalis*^2^ with RMDS values of 2.0 Å and 2.1 Å, respectively. Furthermore, RahU β-sandwich structure shows structural homology to the superfamily of actinoporins, 20-kDa pore-forming proteins found in sea anemones, with a RMSD value of 2.2 Å when compared to equinatoxin II^31^ or stycholysin II^32^ (Fig. S3). Actinoporin toxic mechanism has been extensively investigated and features oligomerization and pore formation upon binding membranes containing sphingomyelin^33^. A key element that enables pore formation of actinoporin is its N-terminal helix, which is, however, absent in aegerolysins (Fig. S3a).

Despite relatively low sequence identity (< 35%) between RahU and fungal aegerolysins OlyA^4^ and PlyA^5^, location of a few amino acid residues is conserved in all structures (Fig. 2b). Some of these residues, such as Val20, Ile22, Ile60, Thr72, Phe76, Phe88, Trp90, Asn100 and Trp102, are positioned in the mainly hydrophobic core to maintain the integrity of β-sandwich architecture. However, a majority of the conserved non-alanine and non-glycine residues, including Trp6, Ser16, Asn24, Trp29, Lys31, Lys38, Asp39, Glu41, Ser43, Arg65, Asp77, Asp80, Asp91, Pro93, Trp94, Thr99, Thr101, Ser116, Asn119 and Thr129, is solvent-exposed and/or positioned in the loop areas, possibly providing a uniform environment to aid in the membrane lipid recognition step.

In certain crystallization trials, we preincubated RahU with its previously reported ligand lysoPC^24,25^, which we assumed could contribute to the formation and stabilization of protein crystals, and even indicate the interface between RahU and lipids. When lysoPC was used in the RahU crystallization trials, the crystals diffracted beyond 1.13 Å. No electron density could be found to ascribe the lysoPC ligand bound to the RahU, and the structure essentially resembled the apo-RahU structure (Figs. 2c and S2, Table S1). However, we noted some conformational differences between the two structures in the regions of three loops A-C on one side of the molecule (Fig. 2a, c), thereby revealing their flexibility. In addition, a clearly defined additional density was found in the cavity between the slightly displaced B-loop and C-loop, in comparison to apo-RahU structure (Fig. 2c—inset), into which a model of Tris molecule could be nicely fit. It should be noted, that Tris buffer was used in the purification protocol at 20 mM concentration as in the case of ‘apo’ RahU structure, but concentration of Tris was in contrast to apo RahU 100–120 mM in the crystallization condition. A molecule of Tris is accommodated directly via Asp92 and the conserved Asn100 side chains together with the main chain carboxyl or amino groups of Pro93 (also conserved), Lys95, Ala98, Thr121, Gly122 and Thr123 (Fig. 2d). The main chain carboxyl group of the conserved Trp94 is also involved in the Tris binding indirectly via a water molecule. Importantly, mutagenesis of this tryptophan equivalent in PlyA2 from *P. eryngii* and OlyA from *P. ostreatus* exhibited decreased binding ability toward vesicles containing sphingomyelin^4,9^, suggesting a key role in initial membrane contact similarly to Trp112 in actinoporins^34^. Yet, by comparing RahU-Tris complex to the OlyA-sphingomyelin complex^4^, and sticholysin II structure in complex with phosphocholine^32^, we noticed that the Tris molecule is positioned on the other side of the β-sandwich (Fig. S3b). Although not conclusive, RahU-Tris complex suggests an alternative binding site for lipids in RahU.

### Tris inhibits RahU binding to CPE

**Figure 3:**
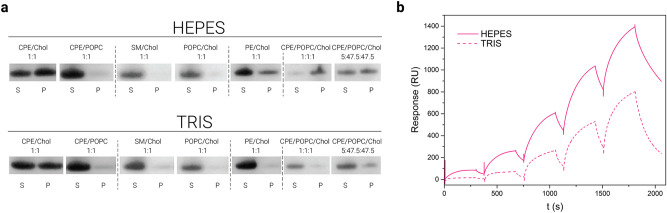
Binding analysis of RahU protein to CPE-enriched lipid vesicles. (**a**) Binding of RahU protein to multilamellar vesicles with various lipid compositions, as indicated with mole-to-mole ratios. The liposome-RahU co-sedimentation experiments were performed in either Hepes or Tris buffer as marked above the gels. The supernatant (S) and pellet (P) fractions were resolved on SDS-PAGE gels and stained with Coomassie Blue. For ease of comparison, results obtained in Hepes buffer and presented in Fig. 1a are shown also here. The dotted vertical dividing lines indicate the cropped parts of the same gel image. The full-length gels are shown in Fig. S1. (**b**) Binding of 4 µM RahU, either in 10 mM Hepes (solid line) or in 20 mM Tris buffer (dotted line) to the large unilamellar vesicles composed of equimolar CPE/POPC/cholesterol. Vesicles were immobilized to Biacore L1 chip to 9500–9600 RU and analytes were injected for 300 s at 10 μL/min flow rate. To show only the RahU-vesicle interaction, the baseline was set as 0 RU after vesicle immobilization. For ease of comparison, results obtained in Hepes buffer and presented in Fig. 1b are shown also here. Representative sensorgrams from two independent experiments are shown. *S* supernatant, *P* pellet, *CPE* ceramide phosphorylethanolamine, *Chol* cholesterol, *POPC* 1-palmitoyl-2-oleoyl-*sn*-glycero-3-phosphocholine, *SM* sphingomyelin, *PE* phosphatidylethanolamine.

To find whether the binding of Tris to the cavity in RahU indicated the binding of a lipid receptor, we checked the effect of Tris on the RahU binding to CPE. We performed sedimentation and SPR assays to measure the binding of RahU in either Tris (7.4) or in Hepes (7.4) buffer to the vesicles of different lipid composition (Figs. 1, 3, S1). Comparison of the sedimentation assays in the presence of Tris or Hepes showed that Tris to some extent reduces the interaction of RahU with CPE- and phosphatidylethanolamine-containing membranes (Figs. 3a, S1). The comparison of the two SPR sensorgrams shows that Tris indeed interferes with RahU binding to the membranes (Fig. 3b).

**Figure 4:**
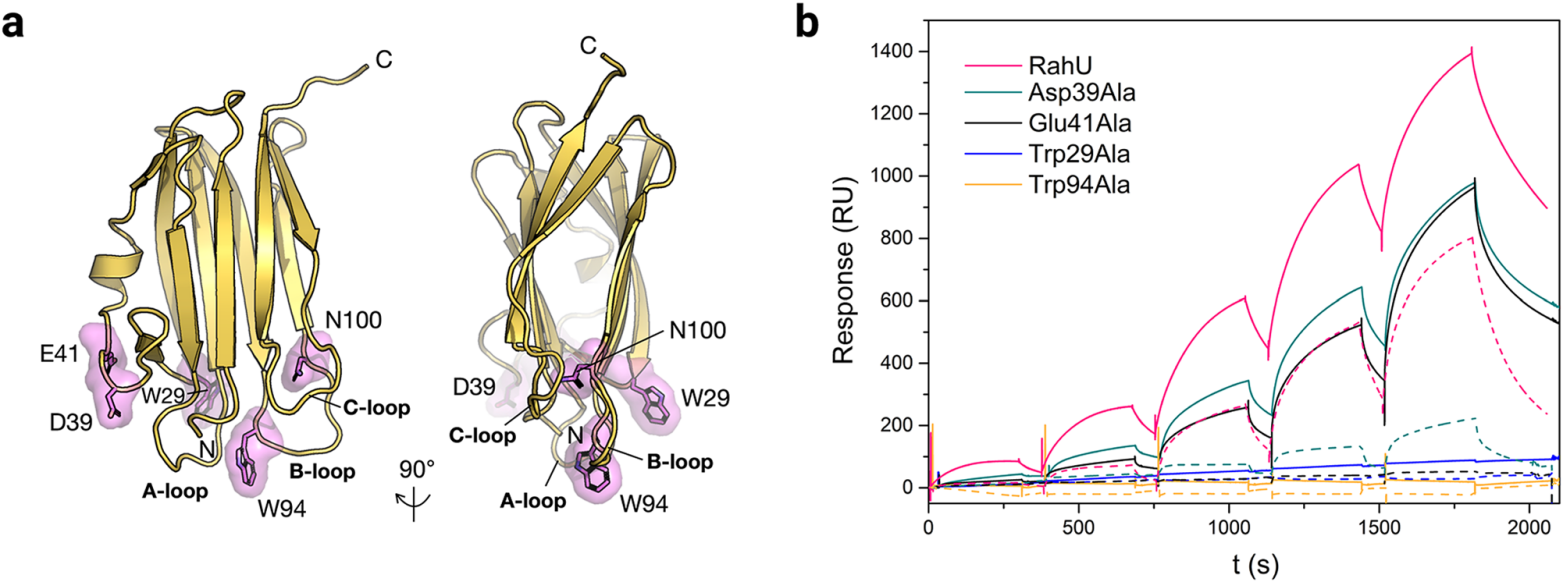
Interaction of RahU single alanine substitution derivatives with the CPE-enriched lipid vesicles. (**a**) RahU structure with marked residues substituted by alanine. (**b**) Binding of single alanine substitution RahU derivatives (4 µM) to the equimolar or CPE/POPC/cholesterol vesicles, either in 10 mM Hepes (solid line) or in 20 mM Tris buffer (dotted line). The vesicles were immobilized to Biacore L1 chip to 7000–11,000 RU and the analytes at 4 µM were injected for 300 s at 10 μL/min flow rate. To show only the RahU-vesicle interaction, the baseline was set as 0 RU after vesicle immobilization. Representative sensorgram from two independent experiments are shown.

To better characterise RahU-membrane interactions, we prepared single alanine RahU mutants carrying a modified residue in the proximity of the Tris-binding cavity (Figs. 4a, S4 and S5) and investigated the binding of the RahU mutants with the CPE-enriched membrane. We selected the residues Trp94 and Asn100, both positioned in the loop that forms contacts with Tris in complex with RahU (Figs. 2c and 2d). Furthermore, to study the effect of a surface-exposed residues at the site opposite the Tris binding site (loops A-C), we mutated the residues Asp39 and Glu41. In addition, since tryptophan residues were previously shown to be important for protein-membrane interaction^35,36^, we also modified the surface-exposed Trp29 (Fig. 4a). The far-UV circular dichroism (CD) spectra and the tryptophan fluorescence spectra of the mutated proteins, with the exception of RahU-Asn100Ala, were comparable to the spectra for the wild type protein, implying that the amino acid substitutions did not noticeably interfere with the mutants' structures (Figs. S6 and S7). Since the RahU-Asn100Ala mutant was most likely in a non-native form, we did not include this mutated protein in further studies. In comparison to the wild type protein, as determined by SPR in Hepes buffer, the RahU-Trp29Ala or RahU-Trp94Ala derivatives exhibited no binding to the equimolar CPE/POPC/cholesterol even at a protein concentration of 4 µM, which proves that these two residues on the RahU surface are important for membrane binding (Fig. 4b). The substitution of either Asp39 or Glu41 with alanine impaired the binding of these RahU variants to the vesicles, but these two residues do not appear to be crucial for RahU interaction with the membranes (Fig. 4b). When the interaction of these two mutant proteins with the vesicles was studied in Tris buffer, we observed that Tris inhibited binding of 4 µM RahU-Asp39Ala and blocked 4 µM RahU-Glu41Ala interaction with CPE/POPC/cholesterol membrane (Fig. 4b). Therefore, in addition to the RahU tryptophan residues at positions 29 and 94, which are required for the binding of RahU to CPE-containing membranes, the Tris-binding pocket, a narrow RahU cavity flanked by the B and C loops (Fig. 2c), is also important for establishing the interaction of the RahU molecule with CPE/cholesterol membranes.

### *P. aeruginosa* RahU interacts with the cytoplasmic membrane of Sf9 insect cells

**Figure 5:**
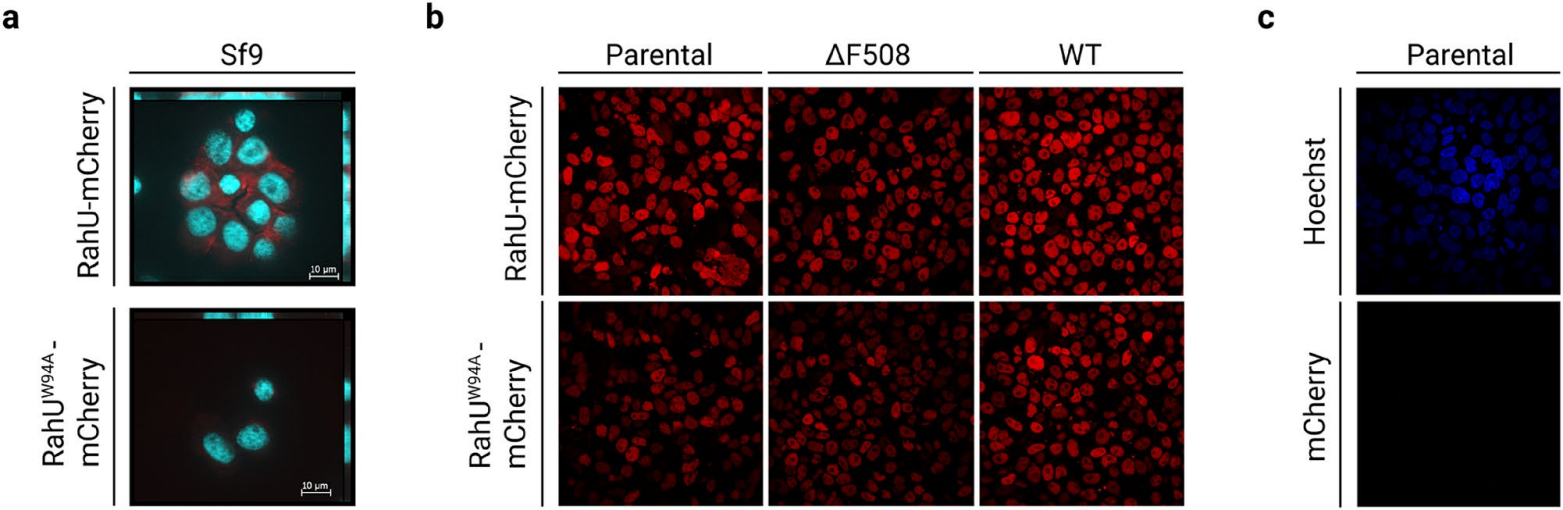
Analysis of RahU interaction with the host cells. Representative fluorescence images depicting (**a**) combined signal of mCherry (red) and DAPI stained nuclei (blue) in fixed *Spodoptera frugiperda* (Sf9) insect cells, after exposure to 10 µM RahU-mCherry (above) or RahU-Trp94Ala-mCherry (below), showing absence of the signal in the latter. (**b**) Fixed HEK293 cells stained with RahU-mCherry (upper frames) and RahU-Trp94Ala-mCherry (below), showing signal in nuclei of Parental cells not expressing CFTR and serving as negative control (left column), ΔF508 HEK293 cell line with CFTR mutation causing cystic fibrosis and wild type HEK293 cell line expressing CFTR (right column). (**c**) Fixed HEK293 cells depicting blue Hoechst33342 signal in nuclei (upper frame) and absence of red signal after mCherry staining (below).

We previously showed that fungal aegerolysins fused to a C-terminal mCherry tag, OlyA6-mCherry and EryA-mCherry from the genus *Pleurotus* and NigA2 from *A. niger* interact with membranes of CPE-containing Sf9 insect cells^10,13,15^. Therefore, we performed microscopy experiments to study RahU interaction with the insect cells Sf9 (Fig. 5a) in comparison to human embryonic kidney (HEK293) cells containing sphingomyelin as main membrane sphingolipid (Fig. 5b). Since *P. aeruginosa* is an important opportunistic human pathogen associated with chronic lung infections in cystic fibrosis patients^37^, we also analysed whether RahU can interact with the HEK293 cell line lacking or stably expressing the cystic fibrosis transmembrane conductance regulator (CFTR), a key factor of cystic fibrosis incidence^38^. We used RahU-mCherry, and its derivative RahU-Trp94Ala-mCherry, in these two experiments.

The fluorescent microscopy data obtained in the assay showed the binding of RahU-mCherry to Sf9 insect cells, but not to HEK293 cells (Figs. 5a, b). For the latter cell line, we show that it lacks, or contains negligible concentration of CPE in the membranes (Fig. S8). Expectedly, the RahU mutant, RahU-Trp94Ala, that is not able to recognize CPE-enriched membranes, exhibited negligible interaction with membranes of both cell lines studied. Unexpectedly, we observed that RahU–mCherry or the mutated RahU-Trp94Ala–mCherry protein (Fig. 5b), but not the mCherry alone (Fig. 5c), localizes to the nucleus of the formaldehyde-treated human cell lines HEK293, with or without CFTR (WT or ΔF508) expression. Further experiments could clarify whether *P. aeruginosa* can deliver RahU into human cells and which processes RahU might affect.

To confirm that RahU interacts with the cell lipids of insects and not with other membrane components, we corroborated the results of fluorescence microscopy by blotting the lipids extracted from Sf9 cells on the membranes and applying fluorescence-labeled RahU to assay the interaction (Fig. S9). In addition, we tested the interaction of RahU with commercial lipids. Our results show binding of RahU-mCherry to the commercial mixture of CPE and cholesterol (molar ratio 1:1) and no binding of RahU either to pure commercial CPE or cholesterol. These findings imply that this interaction requires the combination of both lipids together with a specific cholesterol-bound CPE conformation (Fig. S9). It is suggestive that cholesterol, or some other lipid molecule acting similarly to cholesterol, may induce a conformational change of CPE and thus facilitate the interaction of RahU with Sf9 membranes. A similar mechanism was recently observed for OlyA6 interacting with Sf9 membranes^15^.

To conclude, here we report that *P. aeruginosa* RahU aegerolysin interacts with artificial membranes containing CPE, which is promoted by cholesterol. So far, RahU is the only known CPE-binding aegerolysin of bacterial origin. This interaction seems to be relevant as we determined that RahU binds Sf9 insect cells. The structures of RahU, in the absence and presence of Tris were determined at 1.27 Å and 1.13 Å resolution, respectively, and reveal a typical aegerolysin fold with a central β-sandwich flanked by a loop containing a short α-helix. The Tris molecule is bound to a short channel formed by two flexible loops near the amino-terminal end. Based on the results presented in this study this site is important for the CPE interaction in the membranes of the host organism. At present, it is not clear whether the Tris-binding site is directly involved in the interaction with CPE or Tris plays a role of an allosteric inhibitor preventing conformational changes of the CPE-binding loops. Our data show that RahU tryptophan residues, W29 and W94, which are located in the proximity to the Tris-binding cavity, are required for interaction with the CPE-containing membrane. We show that RahU interacts with the cell membrane of the insect and appears to interact with one or more unidentified target(s) within the nucleus of the human cell line HEK293, expressing either the wild-type CFTR or its non-functional variant. Therefore, our results highlight the need for further experiments to elucidate the biological relevance of RahU in insects and other organisms with membrane CPE^14^. Notably 10 most important plant pathogenic bacterial species^39^ do not produce aegerolysins. Meanwhile, aegerolysin-producing bacteria such as the genera *Pseudomonas*, *Vibrio*, *Acinetobacter*, *Photorabdus*, *Bacillus*, *Clostridium* and others (Pfam PF06355) are known to be insect^40,41^ and/or mollusc pathogens^42^. This observation may coincide with the presence of CPE in the membranes of these animal taxa, but not in plants. The observed binding of RahU to intracellular HEK293 cell structures, presumably lacking CPE, and in particular question whether RahU might play a role in *P. aeruginosa* human infections still remains to be solved.

## Methods

### Chemicals

All chemicals used in the present study were from Sigma–Aldrich (USA). Lipids were obtained from Avanti Polar Lipids. Cholesterol, 1-palmitoyl-2-oleoyl-*sn*-glycero-3-phosphocholine, sphingomyelin and 1,2-Diacyl-*sn*-glycero-3-phosphatidylethanolamine were dissolved in pure chloroform, and ceramide phosphorylethanolamine (C_38_H_77_N_2_O_6_P; from brain porcine, Avanti Polar Lipids, USA) in chloroform/methanol (v/v, 9:1), prior to use.

### Cells

Sf9 cells of *Spodoptera frugiperda*, used for binding analysis, were a generous gift from Dr. Miha Pavšič from Faculty of Chemistry and Chemical Technology, University of Ljubljana, Slovenia. *Escherichia coli* DH5α and BL21(DE3) expression strains were obtained from Novagen, USA. HEK293 cells, and derived HEK293 cells stably expressing ΔF508-CFTR or WT-CFTR were cultured in Dulbecco’s Modified Eagle’s Medium/F12 containing 10% FBS and 1% Penicillin/Streptomycin and maintained in a 5% CO_2_ incubator at 37°C.

### Purification of RahU, RahU-mCherry and their single alanine derivatives for interaction studies with membranes or cells

We modified the *rahU* gene cloned under the control of T7 promotor in the pET21c(+) expression vector (Novagene)^24^, with *Nde*I and *Xho*I restriction sites, to express the RahU, RahU-mCherry and their derivatives, carrying a single amino acid substitution. Plasmid constructs were generated by Quick-Change II Site-Directed Mutagenesis Kit (Stratagene, USA) and pairs of primers (Table S2). Recombinant proteins 16.12 kDa RahU, RahU-Trp29Ala, -Asp39Ala, -Glu41Ala, -Trp94Ala -Asn100Ala, RahU-mCherry and RahU-Trp94Ala-mCherry were expressed with a carboxy-terminal thrombin-cleavable hexahistidine (His_6_) tag in the *E. coli* BL21(DE3) expression strain. Cells were grown in lysogeny broth medium supplemented with 100 μg/mL ampicillin at 20°C with shaking until absorbance at 600 nm reached ~ 1. Protein overexpression was induced with 0.5 mM IPTG. The RahU protein and its derivatives were purified by Ni-chelate affinity chromatography (HIS-Select Nickel Affinity Gel, Sigma), as described previously^24^. Purified proteins were dialyzed against 20 mM Tris–HCl, 130 mM NaCl pH 7.4 or 10 mM HEPES, 150 mM NaCl pH 7.4, and stored at –20°C. These proteins were used in the sedimentation and in the SPR studies in order to assay the interaction of the selected protein with the vesicle of specific lipid composition. Protein concentrations were determined by NanoDrop 1000 (Thermo Scientific, USA).

### Preparation of multilamellar and large unilamellar lipid vesicles

Multilamellar vesicles (MLVs), containing a specific molar ratio of selected lipids, were prepared to a final concentration of 5 mg/mL of total lipids. Each lipid film was prepared by evaporation of organic solvents under reduced pressure and hydrated with Tris (20 mM Tris–HCl, 140 mM NaCl, 1 mM EDTA, pH 8.0) or Hepes (10 mM HEPES, 150 mM NaCl, pH 7.4) buffer and vortexed extensively to gain MLVs^43^. Large unilamellar vesicles (LUVs) composed of CPE/POPC/cholesterol (molar ratio, 1:1:1) were prepared from MLVs, by 8-times snap freezing the vesicles in liquid nitrogen, each time followed by thawing at 60°C, and extruded through the polycarbonate membrane with 100 nm pores at ~ 50°C (Millipore, Germany). MLVs and LUVs were stored at 4°C and used within five days.

### Co-sedimentation assay of RahU with multilamellar lipid vesicles

MLVs (5 mg/mL), composed of specific molar ratio of selected lipids, were incubated with RahU at 10:1 (w/w) ratio in Tris (20 mM Tris–HCl, 140 mM NaCl, 1 mM EDTA, pH 8.0) or in Hepes (10 mM HEPES, 150 mM NaCl, pH 7.4) buffer at room temperature for 30 min on a rotary shaker at 600 rpm/min. Vesicle-bound RahU was separated from the unbound protein by centrifugation at 60.000 × *g* and 4°C for an hour. The unbound RahU was precipitated with 100% trichloroacetic acid for 10 min on ice and collected by centrifugation (16.100 × *g*, 10 min, 4°C). The sediments were washed twice with ice-cold acetone and left to dry completely. Samples were resuspended in NuPAGE LDS loading sample buffer, heated to 100°C for 5 min, proteins resolved on 4–12% NuPAGE Novex gels (Invitrogen, Thermo Fischer Sientific, USA) and visualized by Coomassie Blue staining.

### Surface plasmon resonance analysis of the protein-lipid binding

Binding measurements of RahU protein and its mutants to LUVs composed of equimolar CPE/cholesterol, CPE/POPC, SM/cholesterol, POPC/cholesterol, PE/cholesterol or CPE/POPC/cholesterol, or 5:47.5:47.5 (mol:mol:mol) CPE/POPC/cholesterol, were performed at the Infrastructural Centre for Analysis of Molecular Interactions at the Department of Biology at Biotechnical Faculty, University of Ljubljana, on a Biacore T100 apparatus (GE Healthcare, USA) at 25°C^24,44^. LUVs, prepared as described above, were immobilized (1400–11,000 response units) on the second flow cell of the L1 sensor chip (GE Healthcare, USA) for 600 s at 2 µL/min flow rate, while the first flow cell was used as a reference. Both flow cells were blocked with 0.1 mg/mL BSA (60 s, 30 µL/min) to prevent nonspecific interactions of analyte with lipophilic groups, covalently attached to carboxymethylated dextran. For the single cycle kinetics experiment the 10 mM HEPES, 150 mM NaCl, pH 7.4 or the 20 mM Tris–HCl, 140 mM NaCl, 1 mM EDTA, pH 7.4 were used as a running buffer. The RahU protein or its derivatives (0.5, 1, 2, 3 and 4 µM) either in Hepes or Tris buffer were injected across the vesicles at 5 µL/min for 300 s from a low to high concentration with short dissociation times in between and a 180 s dissociation time at the end. The sensor chip surface was regenerated with 0.5% SDS (120 s, 10 µL/min), 40 mM β-D-glucopyranoside (120 s, 10 µL/min) and 30% ethanol (120 s, 10 µL/min). The data were evaluated using BIAevaluation software (GE Healthcare, USA).

### Expression and purification of RahU protein for structural studies

The gene encoding RahU, with the TEV cleavage site followed by the hexahistidine tag to its C-terminus, was inserted into a pET21c vector using *Nde*I and *Xho*I restriction sites. Upon transformation, *E. coli* BL21(DE3) cells were grown in Terrific Broth medium supplemented with 100 μg/mL ampicillin at 37°C with shaking until absorbance at 600 nm reached ~ 1. RahU overexpression was induced with 0.5 mM IPTG. Cells were harvested after 20 h of shaking at 20°C by centrifugation at 4°C and 5647 × *g* for 10 min. Cells were resuspended in the lysis buffer (50 mM Tris/HCl pH 7.5, 250 mM NaCl, 10% (v/v) glycerol). Cell suspension was sonicated and debris was removed by centrifugation at 47,850 × *g*, 4°C for 45 min. The supernatant was filtered sequentially through 0.45 μm and 0.22 μm filters, aliquoted and frozen at − 80°C until use. For protein purification the supernatant was thawed and loaded to Ni-chelate affinity column column (Ni–NTA, Qiagen), washed extensively with 50 mM NaH_2_PO_4_ pH 7.5, 300 mM NaCl, 10 mM imidazole, and the bound fraction was eluted with the same buffer, but now containing 300 mM imidazole. TEV protease was added to a final concentration of ~ 30 μg/mL to the eluted fraction and this solution was dialyzed 1:100 overnight at 4°C against 50 mM NaH_2_PO_4_ pH 7.5, 300 mM NaCl. The TEV-cleaved RahU was loaded onto Ni–NTA column to separate the non-cleaved protein and the His-tagged TEV protease from the TEV-cleaved fraction of RahU. The unbound fraction containing TEV-cleaved RahU was concentrated with Amicon Ultra 10 kDa MWCO (Merck, Germany). This was followed by the final purification step by size exclusion chromatography on Superdex 75 (GE Healthcare, UK), which was equilibrated with 20 mM Tris/HCl pH 7.5, 150 mM NaCl, 5% (v/v) glycerol. Fractions containing purified RahU were pooled and concentrated to 31 mg/mL, aliquoted, flash frozen in liquid N_2_ and stored at − 80°C until use.

### Crystallization, X-ray data collection and structure determination

Crystals of apo-RahU were grown using the sitting-drop vapor-diffusion method from drops composed of 0.1 μL RahU (31 mg/mL or 21 mM) and 0.1 μL reservoir solution (0.2 M di-Sodium tartrate, 20% (w/v) PEG 3350) and equilibrated over 70 μL reservoir solution at 20°C. To generate crystals of lysophosphatidylcholine (lysoPC) bound RahU that later turned out to be a RahU-Tris complex, we first diluted RahU to 1.5 mg/mL (1 mM), added water-solubilized lysoPC in molar ratio 5:1 (lysoPC:RahU) and incubated the mixture overnight using gentle mixing at room temperature. In the following step, the mixture was concentrated to 26 mg/mL RahU concentration and crystallized using the same method as described above, with the reservoir solution containing 0.1 M Tris/HCl pH 8.5, 8% (w/v) PEG 8000, and the final cryoprotectant solution 0.12 M Tris/HCl pH 8.5, 10% (w/v) PEG 8000, 25% (v/v) ethylene glycol.

Diffraction data for both types of crystals were collected at XRD1 beamline at Synchrotron Elettra (Trieste, Italy) and processed by XDS^45^. Phases were obtained by molecular replacement using Phaser^46^, with the crystal structure of aegerolysin from *P. ostreatus* Pleurotolysin A (PlyA) (PDB ID 4OEB) as an initial search model. Crystallographic refinement was done in phenix.refine^47^ and iterative rebuilding in Coot^48^.

Apo-RahU and RahU-Tris crystal structures were determined at 1.27 Å and 1.13 Å resolution, respectively (Table S1). Structures were deposited to Protein Data Bank under the PDB-ID 6ZC1 for apo-RahU and 6ZC2 for RahU-Tris complex.

### Sodium dodecyl sulfate–polyacrylamide gel electrophoresis (SDS-PAGE) of RahU or its derivatives used in interaction studies with membranes or cells

Purified RahU-His_6_ and its derivatives were denatured by heating the samples at 95°C for 15 min, along with dithiothreitol (DTT) reducing agent with a final concentration of 180 mM as well as loading buffer NuPAGE LDS Sample Buffer (Invitrogen, USA). Samples were then subjected to NuPAGE Novex 4–12% Bis–Tris Protein Gel (Invitrogen, Thermo Fisher Scientific, USA). SDS-PAGE electrophoresis was carried out at constant voltage 200 V for approximately 50 min followed by Coomassie Blue staining.

### Western blotting of RahU or its derivatives used in interaction studies with membranes or cells

Western blotting of RahU and its derivatives was performed to immunodetect proteins of our interest. Protein transfer from SDS-PAGE gel to polyvinylidene fluoride (PVDF) membrane (Biorad, USA) was performed at a constant current of 150 mA for 90 min, using Mini Trans-Blot Electrophoretic Transfer Cell (Bio-Rad, USA). After an overnight incubation at 4°C in TBS buffer (10 mM Tris, 150 mM NaCl, pH 7.4) containing 4% bovine serum albumin (BSA), the membrane was transferred to TBS buffer containing 4% BSA and primary mouse monoclonal anti-His_6_ tag antibody (Invitrogen, USA) and incubated for 1 h at room temperature with gentle shaking, followed by washing and incubation with the secondary goat monoclonal anti-mouse IgG-HRP antibodies (Thermo Scientific, USA). Unbound antibodies were washed 3 times for 10 min in TBS buffer. After adding peroxidase substrate, bound proteins were detected by chemoluminiscence using G:BOX (Syngene, Great Britain).

### Circular dichroism of RahU or its derivatives used in interaction studies with membranes

To analyse secondary structure conservation of purified proteins, far-UV CD spectra of RahU or its mutants were recorded on a Chirascan CD spectrometer at 20°C (Applied Photophysics, Great Britain), using 0.75 mg/mL of each protein in 10 mM phosphate buffer (pH 8), except for RahU Asn100Ala mutant that was at 0.2 mg/mL concentration. The spectra were measured in the region of wavelength from 190 to 250 nm, using a 1 mm path length quartz cuvette, 0.5 nm bandwidth and 1 s response time. Five successive scans for each spectrum were averaged, processed, baseline corrected, smoothed and converted with Chirascan software (Applied Photophysics, Great Britain).

### Tryptophan fluorescence of RahU or its derivatives used in interaction studies with membranes

Tryptophan spectra of RahU and its mutants were recorded on a spectrofluorimeter FP-750 (Jasco, Japan) using 0.01 mg/mL of each protein in 10 mM Hepes, 150 mM NaCl, pH 7.4 and 1 cm path length quartz cuvette. Due to the tyrosine absence, the tryptophan fluorescence was excited at 280 nm, with emission 300–450 nm wavelength range, 250 nm/min scan speed and 5 nm bandwidth, at 20°C.

### Interaction of fluorescently tagged RahU and its derivative RahU-Trp94Ala with cell membranes of *Spodoptera frugiperda* (Sf9) insect cells

The binding of RahU-mCherry and its single alanine derivative, RahU-Trp94Ala-mCherry, to the membranes of Sf9 insect cells was investigated using fluorescence microscopy. Cells were grown in Insect-XPRESS medium (Lonza, Switzerland) on chambered coverslips (iBidi, Germany) at a final concentration of 300.000 cells/mL. After an overnight incubation at 28°C, cells were washed in a phosphate buffered saline (PBS), fixed with 2% formaldehyde in PBS for 15 min at room temperature and washed again with PBS. For labelling with aforementioned proteins, cells were incubated with 10 µM RahU-mCherry or RahU-Trp94Ala-mCherry for 15 min at room temperature. 10 µM mCherry was used as a negative control. Cell nuclei were labelled with DAPI (4′,6-diamidino-2-phenylindole). Coverslips were examined by a fluorescent microscope AxioImager Z1 supplemented with an ApoTome device enabling optical sectioning, whereas images were acquired using Zeiss AxioCam HRc camera (Carl Zeiss, Germany).

### Interaction of fluorescently tagged RahU and its derivative Trp94Ala with HEK293 cells

HEK293 cells were grown on poly-D-lysine-coated glass dishes. After an overnight culture, cells were fixed with 2% formaldehyde at room temperature for 15 min. After washing with PBS three times, cells were incubated with 4 µM mCherry, RahU-mCherry, and RahU-Trp94Ala-mCherry for 15 min. Cells were washed with PBS and then incubated with Hoechst33342 (5 µg/mL) in PBS for 30 min at room temperature. Cells were washed again with PBS before imaging by the fluorescence microscope.

### Dot-blot assay

Binding of RahU-mCherry to lipid standards and lipid extract from Sf9 cell membranes was performed with a dot-blot assay. Lipid standards (7 µg) and lipid fractions (100 µg) were applied on a 0.45 µm nitrocellulose membrane (Bio-Rad, USA). Membranes were blocked with 5% skimmed milk in TBS for 2 h at room temperature on a rotary shaker, following 2 h incubation with 10 µg/mL of RahU-mCherry. After incubation, membranes were washed three times by TBS for 5 min and examined under a stereo microscope (Leica MZ FLIII, Germany) with CCD camera at 1.0 magnification and time of excitation 2 s.

### Lipid extraction from *Cftr*^+^ and *Cftr*^−^ cells

Lipid extraction from *Cftr*^+^ and *Cftr*^−^ cells was performed according to a modified Folch method^49^ in order to separate polar and non-polar lipids. Briefly, 1 g of each cell pellet was suspended in distilled water/ice cold methanol/chloroform (volume ratio, 3:8:4, respectively). Lipid extraction was performed over night at room temperature on a rotary shaker. The next day the samples were centrifuged for 15 min at 994 × *g* at room temperature to separate supernatant from the sediment, which was extracted again as described above. Water was added to each supernatant, in order to obtain final water/methanol/chloroform volume ratio of 5.6:8:4, respectively. Upper, polar phase was separated from lower, non-polar lipid mixture by centrifugation. Each phase was then dried on rotary evaporator and their mass was determined by weighing, following with dissolution in water/methanol/chloroform (volume ratio, 3:8:4) for polar lipids and methanol/chloroform (volume ratio, 1:1) for nonpolar lipids to a final concentration of 10 mg/mL and stored at – 20 °C.

### TLC analysis of *Cftr*^+^ and *Cftr*^−^ cell membrane lipids

Lipid extracts from *Cftr*^+^ and *Cftr*^−^ cells were separated on aluminium TLC sheets (TLC Silica gel 60 F_254_, Merck Millipore, Germany), using chloroform/methanol/25% ammonia mobile phase with a volume ratio of 65:25:4 for non-polar and 40:40:10 for polar lipids. After drying TLC sheet completely, lipids were detected by primuline and ninhydrine staining and visualized under UV and visible light, respectively.

## Supplementary Information

Supplementary Information
