# Supplementary material for: Crystal structure of RahU, an aegerolysin protein from the human pathogen Pseudomonas aeruginosa, and its interaction with membrane ceramide phosphorylethanolamine
Source: Sci Rep. 2021 Mar 22;11:6572. doi: 10.1038/s41598-021-85956-2 (PMC7985367; doi:10.1038/s41598-021-85956-2)
Supplement: Supplementary file 1 — Supplementary Information [file 41598_2021_85956_MOESM1_ESM.docx]

**Supplementary Material**

**Crystal structure of RahU, an aegerolysin protein from the human pathogen *Pseudomonas aeruginosa*, and its interaction with membrane ceramide phosphorylethanolamine**

Eva Kočar, Tea Lenarčič, Vesna Hodnik, Anastasija Panevska, Yunjie Huang, Gregor Bajc, Rok Kostanjšek, Anjaparavanda P. Naren, Peter Maček, Gregor Anderluh, Kristina Sepčić, Marjetka Podobnik, Matej Butala

|  |  |  |
| --- | --- | --- |

**Supporting Table S1.** **Data collection and refinement statistics (molecular replacement).**

|  | Apo-RahU | | RahU-Tris |
| --- | --- | --- | --- |
| **Data collection** |  |  | |
| Space group | C2 | C2 | |
| Cell dimensions |  |  | |
| *a*, *b*, *c* (Å) | 88.1, 48.0, 40.6 | 88.3, 48.1, 40.4 | |
| α, β, γ (°) | 90.0, 110.3, 90.0 | 90.0, 110.2, 90.0 | |
| Resolution (Å) | 50.00 – 1.27 | 50.00 - 1.13 | |
| *R*_meas_ (%) | 10.4 (65.8) | 4.2 (46.8) | |
| *I* / σ*I* | 11.8 (2.5) | 17.2 (3.0) | |
| CC_1/2_ (%) | 99.6 (80.9) | 99.9 (89.9) | |
| Completeness (%) | 93.6 (75.1) | 98.9 (96.8) | |
| Redundancy | 6.5 (5.0) | 3.3 (3.0) | |
| No. unique reflections | 39,475 | 59,150 | |
|  |  |  | |
| **Refinement** |  |  | |
| Resolution (Å) | 34.64 - 1.27 | 41.5 - 1.13 | |
| No. reflections | 39,469 | 59,138 | |
| *R*_work_ / *R*_free_ (%) | 15.9/16.7 | 13.9/ 15.6 | |
| No. atoms |  |  | |
| Protein | 1058 | 1081 | |
| Ethyleneglycol | / | 36 | |
| 2-amino-2-hydroxymethyl-propane-1,3-diol | / | 8 | |
| Water | 267 | 184 | |
| *B*-factors (Å^2^) |  |  | |
| Protein | 14.2 | 16.8 | |
| Ethyleneglycol | / | 28.6 | |
| 2-amino-2-hydroxymethyl-propane-1,3-diol | / | 19.5 | |
| Water | 30.0 | 21.4 | |
| R.m.s. deviations |  |  | |
| Bond lengths (Å) | 0.005 | 0.005 | |
| Bond angles (°) | 0.7 | 0.8 | |
| Ramachandran plot |  |  | |
| Favored (%) | 98.5 | 98.5 | |
| Allowed (%) | 1.5 | 1.5 | |
| Disallowed (%) | 0.0 | 0.0 | |

Each dataset was collected from a single crystal. Values in parentheses are for the highest-resolution shell (1.34 – 1.27 Å for apo-RahU and 1.19 – 1.13 Å for RahU-Tris).

Supporting Table S2: Primers used for *rahU* mutagenesis.

| **Primer** | **Sequence** | **Reference of source** |
| --- | --- | --- |
| RahU-W29A-f | 5’-AGAACTTGCCCGCTTGCAAGGTGGCGTTGCGG-3’ | This study |
| RahU-W29A-r | 5’-CCGCAACGCCACCTTGCAAGCGGGCAAGTTCT-3’ |  |
| RahU-D39A-f | 5’-GGTGGAAATCTCGTCGGCCTTGTTGGTGTAGCG-3’ |  |
| RahU-D39A-r | 5’-CGCTACACCAACAAGGCCGACGAGATTTCCACC-3’ |  |
| RahU-E41A-f | 5’-TCCTCGGTGGAAATCGCGTCGTCCTTGTTGG-3’ |  |
| RahU-E41A-r | 5’-CCAACAAGGACGACGCGATTTCCACCGAGGA-3’ |  |
| RahU-W94A-f | 5’-GGCGCCGGATTTCGCCGGGTCATCCCAG-3’ |  |
| RahU-W94A-r | 5’-CTGGGATGACCCGGCGAAATCCGGCGCC-3’ |  |
| RahU-N100A-f | 5'-GTGAAGCTCCAGGTGGCGGTGGCGCCGGATTT-3' |  |
| RahU-N100A-r | 5'-AAATCCGGCGCCACCGCCACCTGGAGCTTCAC-3' |  |
|  |  |  |


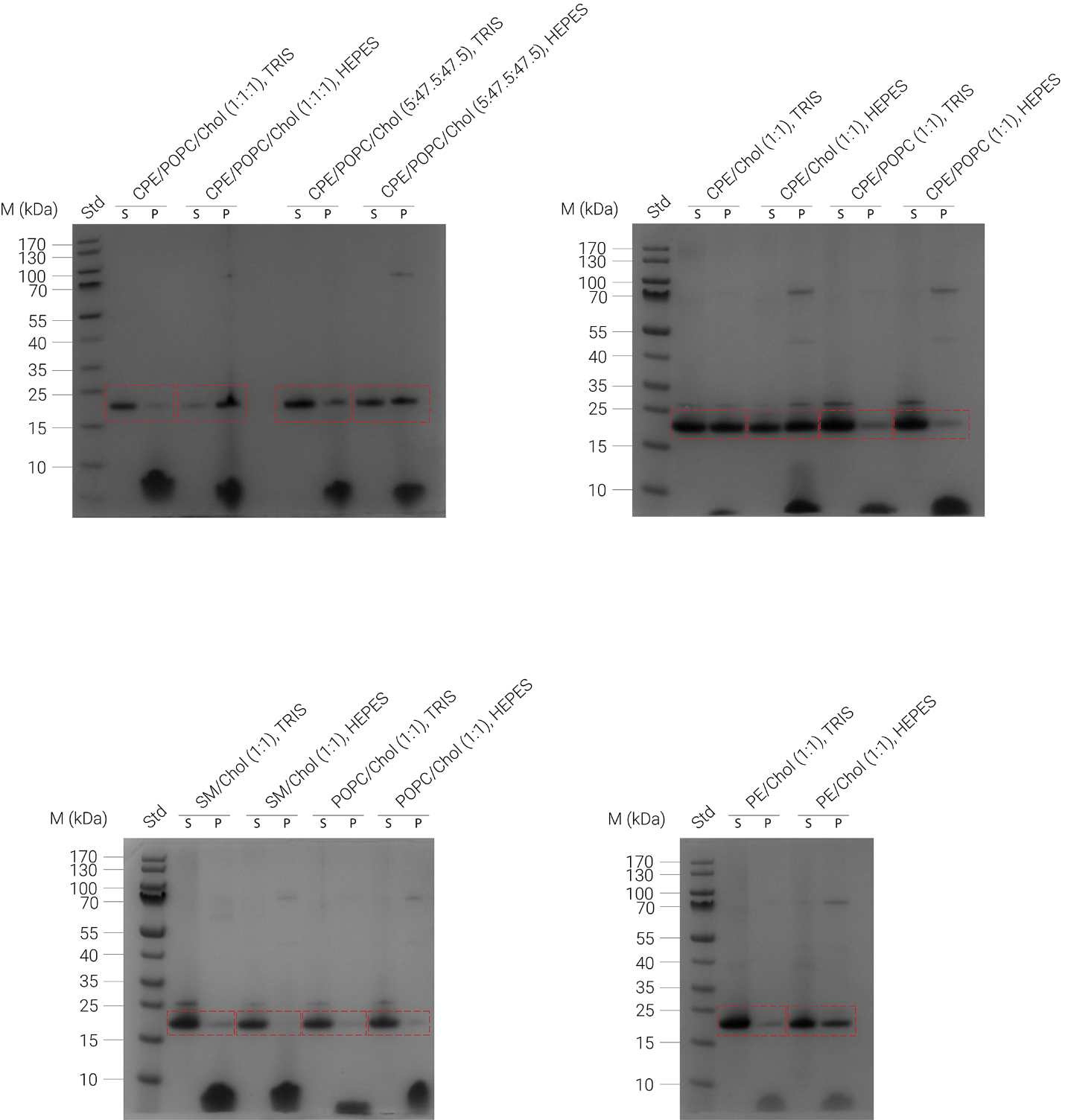


Fig. S1: Binding of RahU protein to multilamellar vesicles with various lipid compositions, as indicated with mole-to-mole ratios. The size of recombinant RahU protein is 16.12 kDa. The dashed rectangles shown in red indicate the areas of the cutout versions shown in Figs. 1a and 3a.

**
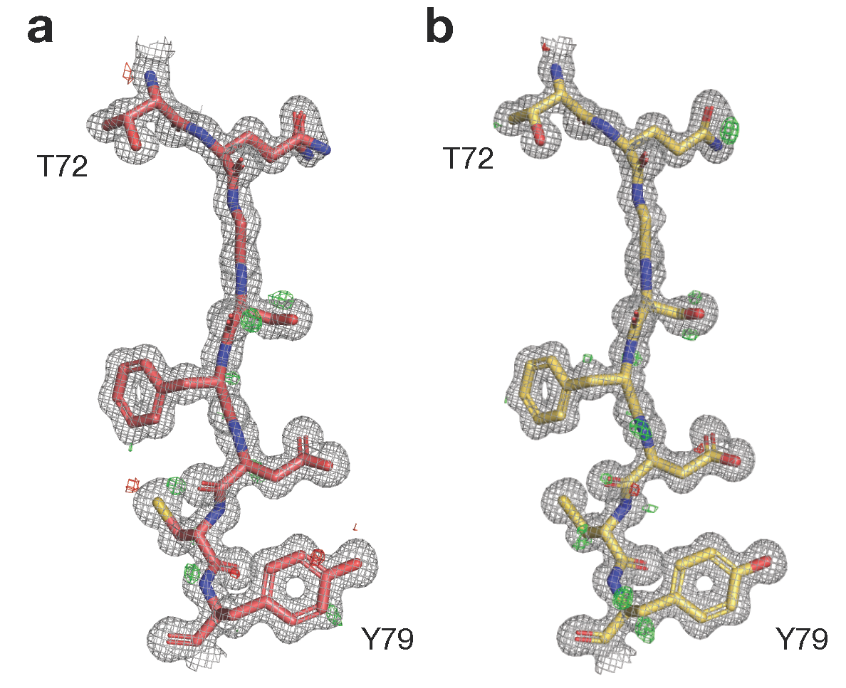
**

**Fig. S2:** Representative 2Fo-Fc electron densities (contoured at 1σ, gray mesh) and Fo-Fc electron density (contoured at 3σ; positive: green mesh, negative: red mesh) of apo-RahU (**a**) and RahU-Tris complex (**b**) polypeptide chain in the region between Thr72 and Asp79.


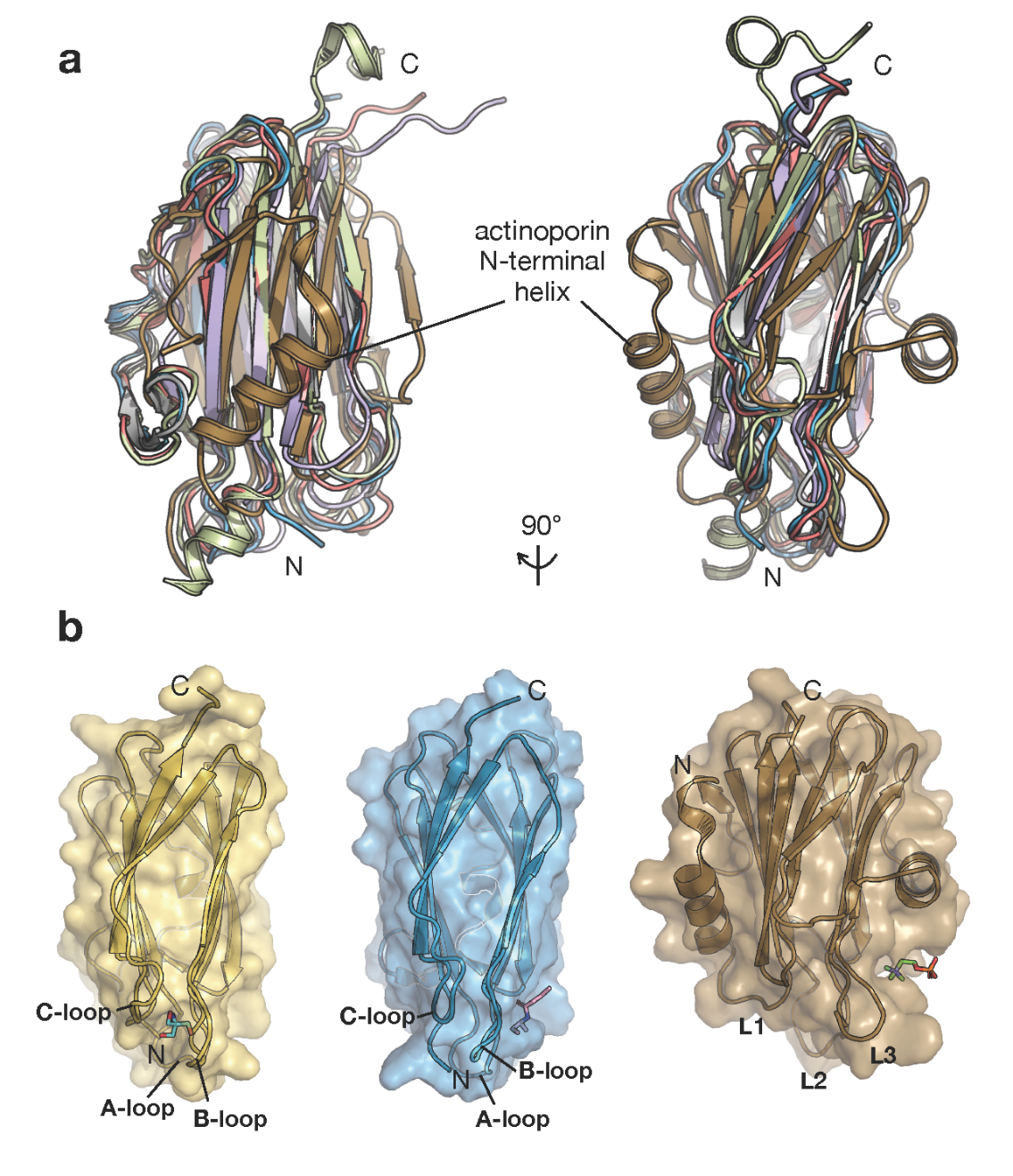


**Fig. S3. Structural comparison of aegerolysin and actinoporin protein families.** (**a**) Superposition of apo-RahU (red), OlyA (PDB-ID 6MYJ; blue), PlyA (PDB ID 4OEB; white), AfIP-1A (PDB-ID 5V3S; green), Cry34Ab1 (PDB-ID 4JOX; violet) and sticholysin II (PDB-ID 1O72, brown). Two orientations, 90° relative to each other, are shown. (**b**) Ligand binding sites in RahU (yellow) in complex with Tris (light blue sticks), OlyA (PDB-ID 6MYJ, blue) in complex with a part of sphingomyelin (pink sticks), and stycholysin II (PDB-ID 1O72, brown) in complex with phosphocholine (green sticks). Loops and N-/C-termini are indicated.


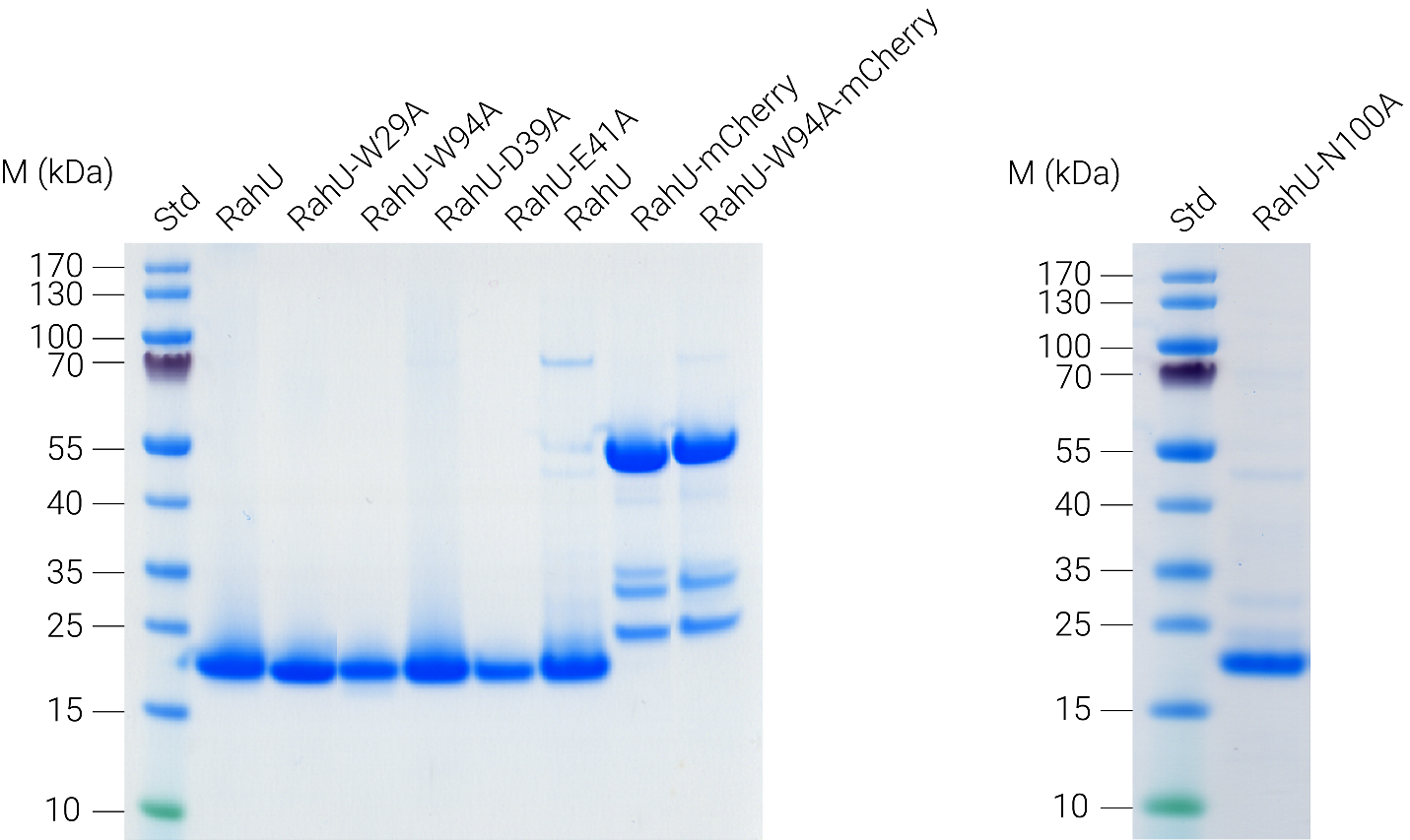


**Fig. S4:** **SDS-PAGE analysis of RahU protein and its mutants.** RahU-His_6_ and its mutants were purified and subjected (7 µg) to NuPAGE^TM^ Novex^TM^ 4-12% Bis-Tris Protein Gel (Invitrogen, Thermo Fisher Scientific, USA). Spontaneously cleaved mCherry-tagged RahU products can be observed. Std, PageRuler Prestained Protein Ladder (Thermo Scientific, USA).


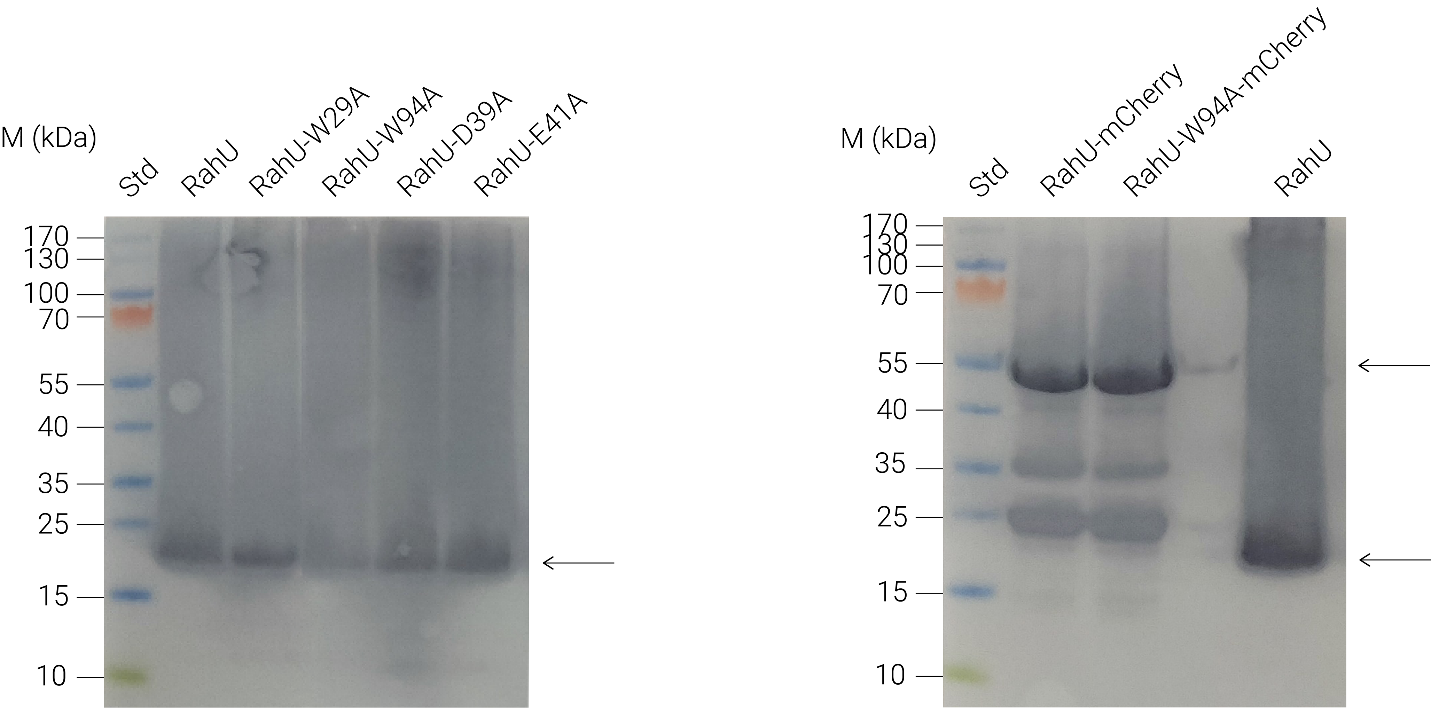


**Fig. S5: Western blot analysis of recombinant RahU mutant proteins**. Western blotting of RahU and its mutants performed to immunodetect proteins of our interest. The arrows mark the position of the RahU (lower arrow) or the RahU-mCherry protein (upper arrow). Std, PageRuler Prestained Protein Ladder (Thermo Scientific, USA).


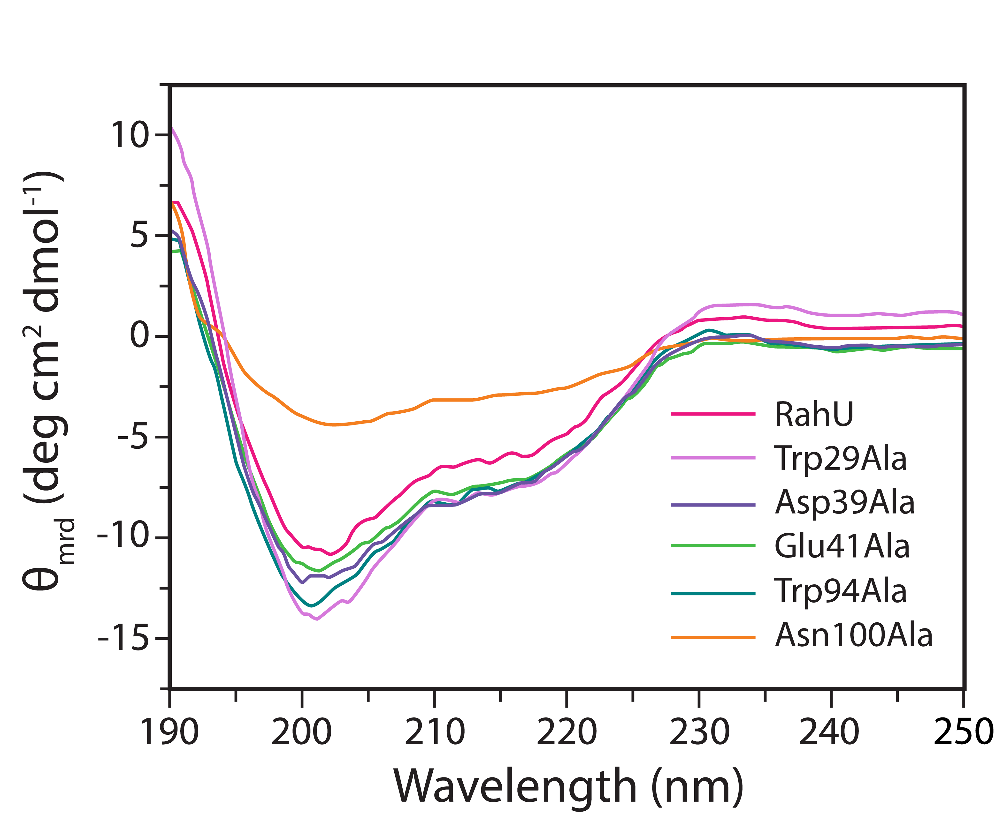


**Fig. S6: Circular dichroism analysis of RahU or its derivatives.** Far-UV circular dichroism spectra of RahU or its derivatives were recorded on a ChirascanTM CD spectrometer. Spectral units were expressed as the mean molar ellipticity per residue.


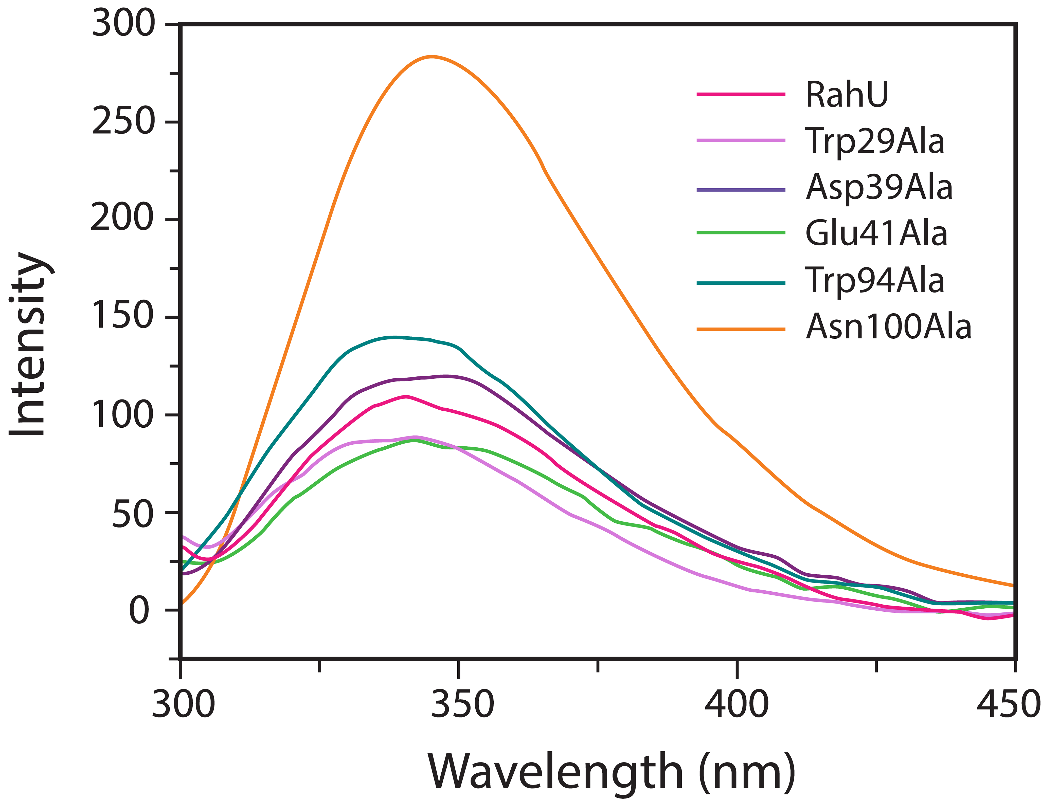


**Fig. S7: Tryptophan emission spectra of RahU and its mutants.** Tryptophan emission spectra of RahU and its mutants were recorded on a spectrofluorimeter FP-750 (Jasco, Japan).


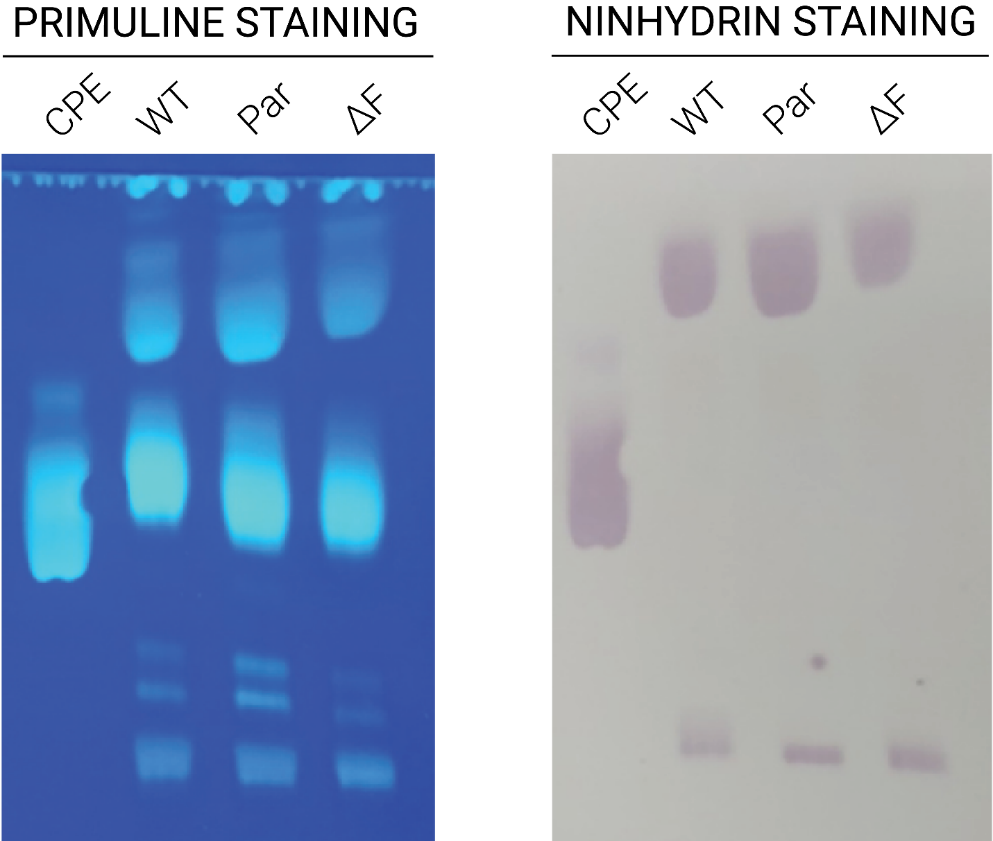


**Fig. S8: Lipid separation of polar and non-polar fractions, obtained after separation of the *Cftr* and *Cftr^-^* cell lipophilic extract**. Lipid separation of polar and non-polar fractions obtained after separation of the *Cftr* and *Cftr^-^* cell lipophilic extract was performed by TLC assay, stained with primuline or ninhydrin and visualized under UV and visible light, respectively. Data obtained imply the absence of CPE in the HEK293 human cell line. CPE, ceramide phosphoethanolamine; Chol, Cholesterol; PE, phosphatidylethanolamine; WT, HEK293; Par, HEK293 *Cftr^-^*; ΔF508, mutant.


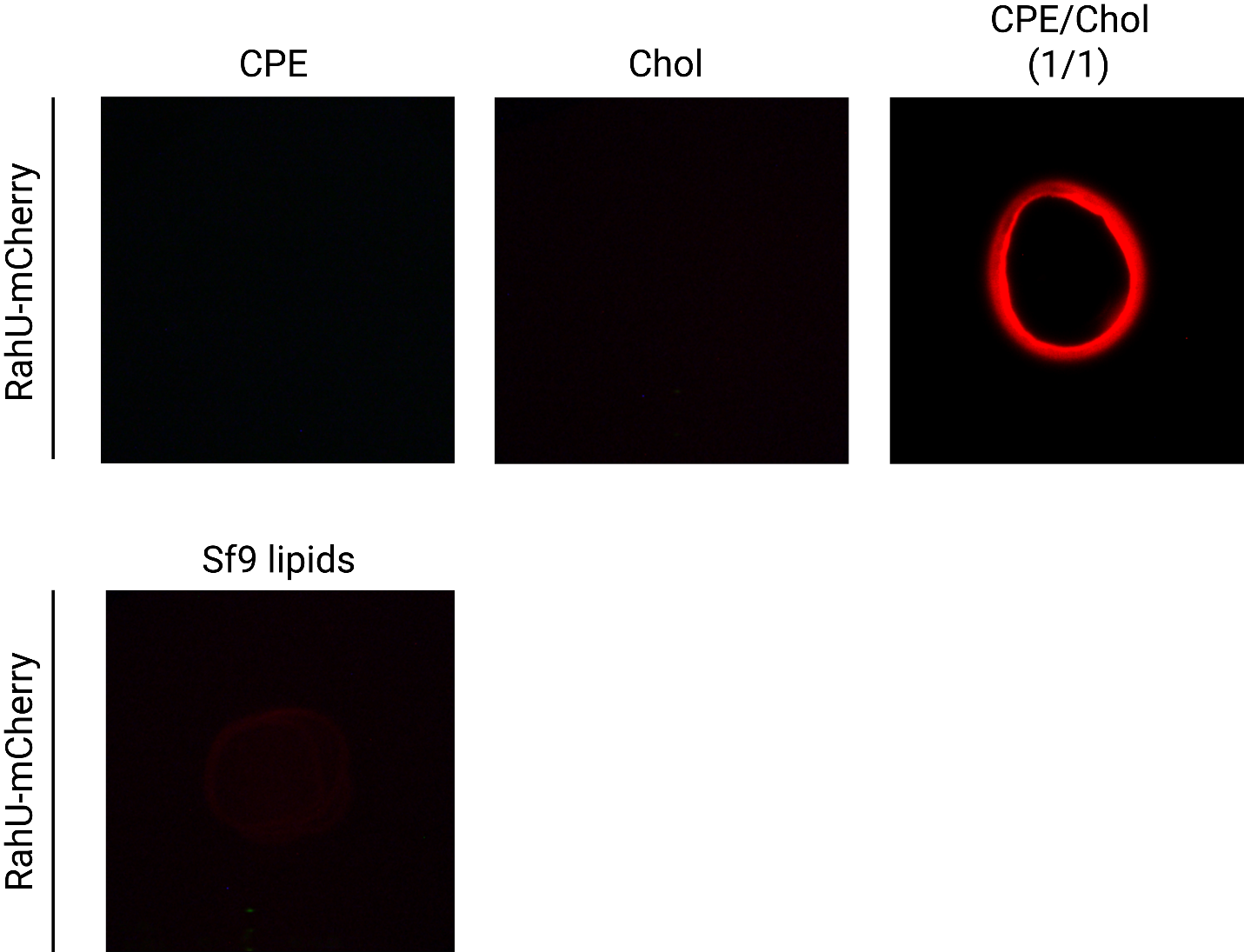


**Fig. S9:** **Interaction of RahU-mCherry with lipid extracts from the *Spodoptera frugiperda* (Sf9) cells.** Lipid blotting of RahU-mCherry to commercial lipids, lipid mixtures and lipids present in polar and non-polar fractions, obtained after separation of Sf9 insect cell lipophilic extract, performed by dot-blot assay. CPE, ceramide phosphoethanolamine; Chol, Cholesterol; PE, phosphatidylethanolamine; Sf9, *Spodoptera frugiperda* cell line.
